# Mycobacterial Etiology of Pulmonary Tuberculosis and Association with HIV Infection and Multidrug Resistance in Northern Nigeria

**DOI:** 10.1155/2013/650561

**Published:** 2013-07-16

**Authors:** Gambo Aliyu, Samer S. El-Kamary, Alash'le Abimiku, Nicholas Ezati, Iwakun Mosunmola, Laura Hungerford, Clayton Brown, Kathleen J. Tracy, Joshua Obasanya, William Blattner

**Affiliations:** ^1^Institute of Human Virology, University of Maryland School of Medicine, 725 West Lombard Street S424, Baltimore, MD 21201, USA; ^2^Department of Epidemiology and Public Health, University of Maryland School of Medicine, Baltimore, MD, USA; ^3^Institute of Human Virology-Nigeria, Plot 252, Herbert Macaulay Way, Central Business District, P.O. Box 9396 Garki, Abuja, Nigeria; ^4^National Tuberculosis and Leprosy Training Center, P. M. B. 1089 Zaria, Kaduna, Nigeria

## Abstract

*Objective*. Data on pulmonary tuberculosis (TB) caused by *Mycobacterium tuberculosis (MTB) complex* in Nigeria are limited. We investigated species of *MTB complex* in TB cases from northern Nigeria. *Methods*. New TB suspects were enrolled, screened for HIV and their sputum samples were cultured after routine microscopy. Genotypes MTBC and MTBDR*plus* were used to characterize the *MTB complex* species and their resistance to isoniazid and rifampicin. *Results*. Of the 1,603 patients enrolled, 375 (23%) had *MTB complex* infection: 354 (94.4%) had *Mycobacterium tuberculosis*; 20 (5.3%) had *Mycobacterium africanum*; and one had *Mycobacterium bovis* (0.3%). Cases were more likely to be male (AOR = 1.87, 95% CI : 1.42–2.46; *P* ≤ 0.001), young (AOR = 2.03, 95% CI : 1.56–2.65; *P* ≤ 0.001) and have HIV (AOR = 1.43, 95% CI : 1.06–1.92; *P* = 0.032). In 23 patients (6.1%), the mycobacterium was resistant to at least one drug, and these cases were more likely to have HIV and prior TB treatment (AOR = 3.62, 95% CI : 1.51–8.84; *P* = 0.004; AOR : 4.43; 95% CI : 1.71–11.45 *P* = 0.002 resp.), compared to cases without any resistance. *Conclusion*. *Mycobacterium tuberculosis* remained the predominant specie in TB in this setting followed by * Mycobacterium africanum* while *Mycobacterium bovis* was rare. The association of TB drug resistance with HIV has implications for TB treatment.

## 1. Introduction

Nigeria has one of the highest burdens for TB in the world and remains a major target in the global control of the disease [1]. In 2011 an estimated 280,000 cases of TB (68% incident cases) were reported from Nigeria which corresponds to a prevalence rate of 280 per 100,000 population according the W.H.O global tuberculosis report of 2012. The incountry prevalence of pulmonary TB due to the *Mycobacterium tuberculosis complex (MTB complex), *particularly species other than* Mycobacterium tuberculosis (M. tuberculosis) *like* mycobacterium bovis* (*M. bovis*) and *Mycobacterium africanum* (*M. africanum)*, [2, 3] is reportedly on the rise. However, this evidence is inconclusive, and data are insufficient on the prevalence of other Mycobacterial species raising question about the importance of the different species of *Mycobacterium tuberculosis (MTB) complex* causing tuberculosis (TB) in Nigeria.

Other pathogenic species of the *MTB complex* group include* Mycobacterium microti (M. microti)* and *Mycobacterium canetti (M. canetti)*, [4] and a recent addition, *Mycobacterium mungi (M. mungi)* [5]. Little is known about the epidemiology of *MTB complex* species associated with pulmonary TB in Nigeria due to limited facilities for TB culture and molecular assays until the recent introduction of U.S President's emergency program for AIDS relief (PEPFAR) and the Global Funds. A better understanding of the circulating *MTB complex* species and their resistance to drugs is essential to guide diagnostic and therapeutic measures aimed at controlling this major public health burden in Nigeria especially with the increase in TB cases due to the prevailing HIV epidemic. Over 3 million people live with HIV/AIDs in Nigeria with a national prevalence of disease estimated at 4.1% in 2010, as released by the country's National Agency for the Control of AIDS (NACA) in its Global AIDS Response Progress Report (GARPR) of 2012.

Pulmonary disease caused by different *MTB complex* species is clinically similar, making surveillance and tracking of specie related to an epidemic a challenge. For example, pulmonary TB caused by *M. bovis *is similar to that caused by *M. tuberculosis* in clinical, pathological, and radiological features [6]. However, in growth media, *M. bovis *tends to have a colony appearance that is distinct from that of *M. tuberculosis* and produces entirely different biochemical reactions including its failure to produce niacin or to reduce nitrate [7]. Conversely, *M. bovis *exhibits striking similarities with *M. africanum* in both morphological appearance and biochemical reactions including its failure to produce niacin or show any positive reaction for nitrate reduction. They both produce similar colonies with poor seeding that may be hard to distinguish [7, 8]. Misclassification errors are therefore likely to occur in environments where *M. bovis* and *M. africanum* are known to coexist. Newer molecular testing techniques are now available for the isolation and characterization of members of the *MTB complex*, including a Genotype MTBC (Hain assay) that enables rapid identification and differentiation of members of *MTB complex* using growth positive samples or direct clinical specimen with higher sensitivity and specificity when compared to conventional methods [9–11].

Identifying the mycobacterial isolate's drug susceptibility pattern through molecular and other techniques is of critical importance in the clinical management of the disease. Resistance to the first line anti-TB drugs, isoniazid, and rifampicin, also known as multidrug resistant TB (MDR-TB), carries a higher risk of death and requires early treatment with second-line drugs [12, 13]. Isoniazid alone resistance produces poor outcomes following treatment with standard TB regimens [14], and isoniazid is frequently used alone or in combination with antiretroviral therapy (ART) for the prevention of TB in HIV-infected subjects, [15–17]. HIV coinfection increases the risk of death by 50 percent among TB cases, [18] with an even higher risk in the presence of MDR-TB [19, 20]. In a study of TB drug resistance and mortality in Peru involving 287 TB patients, 17 of 31 (55%) HIV-MDR-TB patients died before the confirmation of their MDR-TB status [21]. Previous studies from Nigeria reported multidrug resistance among cases of TB receiving treatment with first line tuberculosis drugs in the range of 5% to 19% among new and previously treated patients including those coinfected with HIV [22, 23].

In this study, we utilized a combination of culture and molecular techniques available at the national TB reference center and supported by the PEPFAR program that are fast and sensitive with proven reliability to detect the different species of *MTB complex* causing pulmonary tuberculosis in two TB clinics in Nigeria and to assess the pattern of drug resistance to the top two first-line drugs used in the treatment of pulmonary tuberculosis.

## 2. Methods

This cross-sectional study was conducted at two TB clinics in the state of Kaduna, Nigeria: the National TB and Leprosy Training Center (NTBLTC), in Zaria and the Barau Dikko Hospital (BDH), in Kaduna City, from August 2010 through August 2011. Approvals for the conduct of this study at these sites were granted by the University of Maryland Institutional Review Board and the Nigeria National Health Research Ethics Committee with written expressions of support by the directors of the study sites. 

### 2.1. Settings

 The NTBLTC is the largest TB referral center in northern Nigeria, while the BDH located in the city of Kaduna, is the major referral center within the state. Several hundred patients receive TB/HIV treatment at these facilities. In addition, the NTBLTC serves as the national training center for community health workers and other health personnel involved with tuberculosis detection and treatment at the community level. This center has one of the two national TB reference laboratory that is equipped with a state-of-the-art TB biosafety level 3 (BSL-3) and TB molecular diagnostic laboratories.

### 2.2. TB Detection

Suspected cases with symptoms suggestive of TB and unknown HIV status visiting the facilities for the first time were enrolled. For each patient, a supervised spot sputum sample was collected in the clinic, and HIV status was determined by a serial rapid assay algorithm consisting of Trinity Biotech Unigold and Abbott Determine. Two additional sputum samples were collected the next day (unsupervised early morning sample at home and a supervised sample at the clinic) according to the national guideline at the study time. Current guideline requires only spot and early morning samples. Self-reported data was collected on prior TB treatment, diabetes mellitus, alcohol intake, and cigarette smoking.

The early morning sputum samples were incubated in liquid Mycobacterium Growth Indicator Tubes (MGIT) in the automated BACTEC MGIT 960 machine (Becton Dickinson Diagnostic Instrument Systems) which monitors growth. Samples that failed to show any growth after 42 days of incubation were removed and classified as negatives based on the manufacturer's protocol. Cultures were considered positive for *MTB complex* if they showed a positive growth on the MGIT, presence of acid fast bacilli (AFB) by Ziehl-Neelsen (ZN) stain, and tested positive on a rapid TB antigen assay (SD-Bioline Ag MPT64 Rapid assay; Standard Diagnostics, Kyonggi-do, Korea). The culture confirmed *MTB complex* samples were then characterized with genotype MTBC test (Hain Lifescience, Nehren, Germany) to further identify the different *MTB complex* species. Cultures with positive MGIT growth but negative for *MTB complex* on SD-Bioline were considered non-tuberculous mycobacterium isolates and were excluded from further testing.

To check the ability of the Genotype MTBC assay to distinguish species with few IS*6110* copies (*M. bovis and M. africanum*) from *M. tuberculosis* in this setting, spoligotyping assay was performed on blinded DNA extract from a subset of the *MTB complex* positive specimens by our collaborators at the Institute of Social and Preventive Medicine, Bern, and the Swiss Tropical and Public Health Institute, University of Basel, Basel, Switzerland.

### 2.3. Detection of Resistance to Isoniazid and Rifampicin

Cultures confirmed as containing *MTB complex* were assayed for evidence of resistance to isoniazid and rifampicin with Genotype MTBDR*plus* (Hain Lifescience, Nehren, Germany). This line probe assay was performed according to the manufacturer's protocol as previously described [24]. The DNA strip had a total of 27 reaction zones, of which 21 zones probed mutations and the remaining 6 were control probes for verification of the assay procedures. The control probes consisted of a conjugate control, and amplification control, an *MTB complex*-specific control, an *rpoB *amplification control, a *katG *amplification control and an *inhA *amplification control. Rifampicin resistance was marked by the *rpoB *gene, while isoniazid resistance was marked by the *katG *and *inhA* genes. 

Resistance to rifampicin was identified by the absence of at least one of the wild-type bands or the presence of bands in the region of the *rpoB *gene. Similarly, the absence of at least one of the wild-type bands or the presence of bands suggestive of mutation in either *katG *or *inhA* genes or both identified resistance to isoniazid. Joint occurrence of characteristic features for resistance to both drugs indicated the presence of MDR-TB. A sample in which all the wild-type probes of a gene were present and there was no band suggestive of mutation within the region examined was considered sensitive to the respective drug. Bands in all the six control zones were required to appear correctly; otherwise, the result was considered invalid.

### 2.4. Statistical Analysis

Frequency distributions and proportions of *MTB complex* species, mycobacterial resistance to isoniazid and rifampicin, baseline demographic and related covariates were examined in univariate analyses. Differences in proportions between the categorical groups were evaluated using Chi-square or Fisher's exact test to determine significance of associations between the groups. Two sided *P-*values of 0.05 or less were considered statistically significant. Potential confounders and effect modifiers were checked in stratified analyses. The potential confounders were then added one-by-one to the simple model consisting of the outcome of interest and the predictor covariate. A covariate was retained in the model if it was significant (*P* < 0.05) or if it was considered an important covariate due to biologically plausible relationships. Statistical analysis software (SAS Institute, Inc., Cary, NC, USA) version 9.2 was used for the analysis. 

## 3. Results

A total of 1,603 participants were enrolled with a mean age of 37 years (standard deviation [SD]:13.8 years); males were 897 (56%). The mean body mass index was 19.2 (SD: 4.6). Participants were mostly of the majority Hausa-Fulani ethnic group 1,252 (78%) who occupy northern Nigeria. About 437 (27.3%) engaged in livestock (cattle) farming and within the livestock farming group, 254 (58.1%) spent an average of one hour or more a day tending to their livestock. Most of the participants 1,272 (79.4%) consume milk, or meals prepared with milk, produced locally from livestock on a regular basis. There were 378 (23.6%) participants with positive HIV tests based on the standard of care screening algorithm.  Table 1 provides baseline demographics and some risk factors for *MTB complex* infection among the study participants.

Of the 1,603 participants enrolled, 375 (23.4%) were infected with *MTB complex* species, and, of those, 101 (26.7) had a coinfection with HIV and 91 (5.7%) had pulmonary infection due to organisms found to be acid fast bacilli and culture positives but negative for the MPT 64 antigen on the SD-bioline test (these were considered nontuberculous mycobacterial infections (NTM)) while samples from 234 patients (14.6%) had other bacterial growth that was determined to be contaminants. The MPT 64 antigen negatives (NTM) and the contaminated samples were removed from the analyses; the remaining 903 samples were from clinically symptomatic patients but had no laboratory evidence of mycobacterial infection. Among the *MTB complex* cases identified, 354 (94.4%) were infected with *M. tuberculosis*; 20 (5.3%) had *M. africanum *while one (0.3%) was a case of *M. bovis *infection. Spoligotyping assay performed on blinded DNA extracts from 272 *MTB complex* positive samples obtained by the Genotype MTBC Hain molecular line probe assay revealed a 96% agreement in the frequencies of *M. tuberculosis* and *M. africanum species* between the two assays. No additional cases of *M. bovis* were identified. However, as previously reported [25, 26] from Nigeria, we found 183 (67%) of the isolates to belong to the Latin American Mediterranean (LAM) Cameroon clade family lineage. 

### 3.1. Characteristics of Cases with *MTB Complex* Species  Infection

Within the *MTB complex* cases a comparison between those with *M. tuberculosis* and *M. africanum *infections failed to show any significant difference in demographic or risk factors evaluated. Since there was only one case of *M. bovis, *it was not possible to make comparisons with other groups. Compared to cases without any evidence of mycobacterial infection, *MTB complex* infected cases were more likely to be males (adjusted odds ratio [AOR] = 1.87, 95% confidence interval [CI]: 1.42–2.46; *P* ≤ 0.001), younger than 35 years of age (AOR = 2.03, 95% CI: 1.56–2.65; *P* ≤ 0.001), and have coinfection with HIV (AOR = 1.43, 1.06–1.92; 95% CI: *P* = 0.032) (Table 2).

### 3.2. Pattern and Correlates of Resistance to Rifampicin and Isoniazid

Overall, 23 (6.1%) cases had resistance to at least one of the two drugs (any resistance); of those, 13 (3.5%) cases had resistance only to isoniazid; 5 (1.3%) had resistance only to rifampicin while the remaining 5 (1.3%) cases had resistance to both drugs (MDR-TB). Twenty-two (95.7%) of the cases with any resistance had *M. tuberculosis* infection while the only remaining case of isoniazid resistance had *M. africanum* infection. The risk of resistance, however, was no different between those with *M. tuberculosis* versus *M. africanum*.

Cases with any resistance were more likely to be coinfected with HIV with 12 patients (52.2%) being coinfected (OR: 3.22; 95% CI: 1.40–7.63, *P* = 0.008) and to report prior TB treatment compared to cases without any resistance (OR: 3.81; 95% CI: 1.49–9.45, *P* = 0.008) (Table 3). The odds of a positive history of diabetes mellitus among cases with any resistance were 3.59 times higher than cases without any resistance, but only marginally significant (95% CI: 0.91–13.53,  *P* = 0.079). When isoniazid only resistant cases were compared to cases without any resistance, cases with resistance to isoniazid were more likely to have coinfection with HIV (OR: 3.44; 95% CI: 1.12–10.53, *P* = 0.047) and to report prior TB treatment compared to cases without any resistance (OR: 4.45; 95% CI: 1.38–14.26, *P* = 0.018). 

Repeating this analysis with rifampicin only resistant cases versus nonresistant cases with respect to the measured covariates did not produce any statistically significant associations. However, when comparing MDR-TB cases to cases without any resistance, a trend of associations was observed, where those with MDR-TB were more likely to report alcohol intake and history of diabetes mellitus (OR: 7; 10; 95% CI: 1.19–43.55, *P* = 0.042 and OR: 15.89; 95% CI: 2.51–102.90, *P* = 0.018, resp.) but less likely to belong to the Hausa-Fulani ethnic group (OR: 0.14; 95% CI : 0.01–0.72,  *P* = 0.016). 

The trends observed with isoniazid, rifampicin, and MDR-TB only groups were not adjusted due to small sample sizes. Adjustment was made for the associations involving the larger (any resistance) group, where, after controlling for prior treatment, diabetes mellitus, and ethnicity in a multivariable logistic regression analysis, the odds of HIV coinfection remained significantly higher among cases with any resistance (AOR: 3.62; 95% CI: 1.51–8.84, *P* = 0.004). Likewise, after controlling for diabetes mellitus, ethnicity, and HIV, cases with any resistance were more likely to report prior TB treatment compared to cases without any resistance (AOR: 4.43; 95% CI: 1.71–11.45 *P* = 0.002).

## 4. Discussion

 The burden of pulmonary tuberculosis among suspected cases of TB in this study is high and underscores the relevance of existing TB treatment strategies. The near absence of pulmonary infection by *M. bovis* in our study, and the high frequency of infection by *M. africanum* compared to previous reports [2, 3] indicates a possible change in the distribution of these species or better diagnostic tools with *M. africanum* becoming more relevant for pulmonary TB prevention and control in Nigeria. The shared similarities between *M. bovis* and* M. africanum* often make accurate differentiation harder with conventional methods. The two exhibit striking similarities in both morphological appearance and biochemical reactions and produce similar colonies that may be hard to distinguish [7, 8].

Our findings are, however, in agreement with those of a recently reported study involving samples of subjects from central and southern parts of Nigeria which found a low prevalence of *M. bovis* (1%) and a relatively high prevalence of *M. africanum* (13%) [27]. Other studies from the neighboring west African countries of Ghana, Mali, Cameroun and Burkina Faso [28–31] have also reported a similar trend with very low or absent *M. bovis* (3% from Ghana, 0.8% from Mali, 0.2% from Cameroun, and none from Burkina Faso) and high proportions of *M. africanum* (9 to 28%). The very low prevalence of *M. bovis *in all these studies has further weakened the speculation on the possibility of a human to human airborne transmission of bovine tuberculosis and its relative contribution to new infections in humans [32]. 

The increased frequency of human pulmonary TB due to *M. bovis* reported in other studies [2, 3] was meant to suggest that, in addition to ingestion, an inhalational route of transmission from cattle to human may occur among those working with infected livestock on farms or slaughter houses. Transmission among cattle, however, remains high, with over 95 percent transmission of *M. bovis* occurring through direct contact between cattle. Only 1–5 percent of infected cattle shed the bacteria in their milk, [33] possibly explaining the low transmission to humans. 

Despite the high burden of TB in Nigeria [34–36] and the reported high level of resistance to first line TB drugs [22, 23, 37], our study found the prevalence of resistance to isoniazid, rifampicin, or both to be relatively low. Although the mycobacterial species identification and drug susceptibility tests performed in this study were nonconventional, they were however validated [38, 39]. We did not assess resistance to other TB drugs since our original aim was to determine resistance to isoniazid and rifampicin to identify cases with MDR-TB. The prevalence of resistance to the other two first line drugs ethambutol and pyrazinamide may be high in this population as previously reported in some parts of Nigeria [22, 40]. We plan to include these drugs in our future studies at this site. 

Given our findings that HIV coinfected patients had detectable resistance to at least isoniazid, we anticipate a potential increase in the rate of isoniazid resistance acquisition in this high risk group since every 3 in 10 TB cases in our study and in Nigeria are coinfected with HIV [36]. As expected, tuberculosis treatment in the presence of isoniazid resistance alone is less effective than isoniazid-susceptible TB [14, 41]. In HIV coinfected cases every effort should be made to detect isoniazid resistance and replace it with more effective drugs to avoid the development of MDR-TB. 

The preliminary associations of MDR-TB with ethnicity and diabetes mellitus are interesting and deserve closer scrutiny with larger samples. Despite the fewer number of cases and the fact that our study measured a self-reported history of diabetes mellitus, cases with positive history of diabetes mellitus had an increased tendency to infection by mycobacteria resistant to isoniazid, rifampicin, or both (MDR-TB). While the association with ethnicity may likely be due to chance or some behavioral differences between the ethnic groups, the association with diabetes mellitus and alcohol intake was previously reported in patients with abdominal TB [42]. Behaviorally, alcoholics are likely to be poor adherents to treatment compared to nonalcoholics. 

In conclusion, this study found *M. tuberculosis* and for the first time to a lesser extent *M. africanum* to be the most frequent species of *MTB complex* associated with pulmonary infection in this population, and *M. bovis* pulmonary TB was very rare. The higher tendency for mycobacterial resistance to isoniazid and rifampicin among HIV coinfected TB cases and the correlates of resistance to the two most powerful first line drugs indicate the needs for intensified screening of HIV coinfected cases for evidence of resistance to antituberculosis drugs. The low prevalence of resistance to isoniazid and rifampicin provides an opportunity for aggressive strategies to prevent the spread of resistance that could result in greater morbidity and mortality and a greater strain on the healthcare system given the higher cost of second-line anti-TB drugs that are necessary to treat resistant cases. 

## Figures and Tables

**Table 1:** Demographic characteristics and risk factors for infection with species of *Mycobacterium tuberculosis complex* among participants receiving care at two TB treatment sites in northern Nigeria.

Characteristics	*MTB complex* isolated		No mycobacteria isolated		NTM and other bacteria isolated		*P*-values (Chi-square test)
	*N* = 375		*N* = 903		*N* = 325		
	*n*	%	*n*	%	*n*	%	
Age in years							
≤35	262	69.9	488	54.0	160	49.2	<0.001
>35	113	30.1	415	46.0	165	50.8	
Gender							
Male	249	66.4	475	52.6	177	54.5	<0.001
Female	126	33.6	428	47.4	148	45.5	
Body mass index							
≤19.2	249	66.4	457	50.6	166	51.1	<0.001
>19.2	126	33.6	446	49.4	159	48.1	
Education							
≤8th grade	213	56.8	561	62.1	207	63.7	0.120
>8th grade	162	43.0	342	37.9	118	36.3	
Ethnicity							
Hausa-Fulani	298	79.5	723	80.1	246	75.7	0.245
Other	77	20.5	180	19.9	79	24.3	
HIV infection							
Yes	101	26.9	185	20.5	92	28.3	0.004
No	274	73.1	718	79.5	233	71.7	
Livestock faming							
Yes	86	22.9	256	28.3	95	29.2	0.095
No	289	77.1	647	71.7	230	70.8	
Milk livestock							
Yes	24	6.4	76	8.4	29	8.9	0.391
No	351	93.6	827	91.6	296	91.1	
Consume raw milk							
Yes	327	87.2	809	89.6	284	87.4	0.354
No	48	12.8	94	10.4	41	12.6	
Smoke cigarette							
Yes	105	28.0	152	16.8	60	18.5	<0.001
No	270	72.0	751	83.2	265	81.5	
Consume alcohol							
Yes	65	17.4	101	11.2	45	13.9	0.011
No	310	82.6	802	88.8	280	86.1	
History of diabetes mellitus							
Yes	17	4.6	41	4.5	13	4.0	0.918
No	358	95.4	862	95.5	312	96.0	
Site							
NTBLTC Zaria	315	84.0	803	88.9	273	84.0	0.019
BDH Kaduna	60	16.0	100	11.1	52	16.0	

NTM: nontuberculous mycobacterium.

NTBLTC: National TB and Leprosy Training Centre; BDH: Barau Dikko Hospital.

**Table 2:** Multivariable logistic regression analysis for the risk factors of *MTB complex *infection among participants receiving care at two TB treatment sites in northern Nigeria.

Variables	Unadjusted		Adjusted	
	OR	95% CI	AOR^a^	95% CI
Age				
>35 years^1^	Ref			
≤35 years	1.97	[1.53–2.55]	2.03	[1.56–2.65]
Sex				
Female^1^	Ref			
Male	1.78	[1.39–2.29]	1.87	[1.42–2.46]
BMI				
>19.2^1^	Ref			
≤19.2	1.93	[1.50–2.48]	1.85	[1.42–2.40]
HIV				
Negative^1^	Ref			
Positive	1.43	[1.08–1.89]	1.43	[1.06–1.92]
Cigarette smoking				
Never smoke^1^	Ref			
Current/ever smoke	1.92	[1.44–2.55]	1.58	[1.16–2.16]
Site				
BDH Kaduna^1^	Ref			
NTBLTC Zaria	0.65	[0.46–0.92]	0.57	[0.40–0.83]

^1^Reference group; OR: odds ratio; CI: confidence interval; AOR: adjusted odds ratio.

^a^Adjusted for level of education, ethnicity, livestock faming, and alcohol intake.

BMI: body mass index; BDH: Barau Dikko Hospital.

NTBLTC: National Tuberculosis and Leprosy Training Centre.

**Table 3:** Unadjusted analysis comparing cases with resistance to isoniazid alone, rifampicin alone, both (MDR-TB), and those with any resistance to those without any drug resistance among participants receiving care at two TB treatment sites in northern Nigeria.

Characteristics	Isoniazid resistance only			Rifampicin resistance only			MDR-TB resistance			ANY resistance			NO resistance
	*n* = 13			*n* = 5			*n* = 5			*n* = 23			*n* = 352
	*N* (%)	Odds ratio, 95% CI	*P* value	*N* (%)	Odds ratio, 95% CI	*P* value	*N* (%)	Odds ratio, 95% CI	*P* value	*N* (%)	Odds ratio, 95% CI	*P* value	[Reference category]
HIV infection	7 (53.9)	3.44, 1.12–10.53	0.047	3 (60.0)	4.35, 0.71–26.90	0.110	2 (40.0)	2.01, 0.33–12.02	0.605	12 (52.2)	3.22, 1.40–7.63	0.008	89 (25.3)
Prior TB treatment	5 (38.5)	4.45, 1.38–14.26	0.018	1 (20.0)	1.79, 0.18–16.36	0.485	2 (40.0)	4.71, 0.82–29.44	0.122	8 (34.8)	3.81, 1.49–9.45	0.008	43 (12.2)
Majority Hausa-Fulani ethnic group	13 (100)		0.044	4 (80.0)	1.32, 0.12–11.73	1.000	1 (20.0)	0.14, 0.01–0.72	0.016	18 (78.3)	1.20, 0.41–3.20	1.000	266 (75.6)
Female sex	2 (15.4)	1.19, 0.41–3.87	0.768	2 (40.0)	1.30, 0.21–8.02	1.000	1 (20.0)	0.41, 0.13–4.52	0.669	8 (34.8)	1.13, 0.36–2.62	1.000	118 (33.5)
Site A (Zaria)	12 (92.3)	2.29, 0.32–18.23	0.701	3 (60.0)	0.33, 0.01–1.78	0.192	5 (100)		1.00	20 (86.7)	1.35, 0.43–4.49	1.000	295 (83.8)
Alcohol consumption	0 (0.0)		0.137	1 (20.0)	1.22, 0.11–10.76	1.000	3 (60.0)	7.10, 1.19–43.55	0.042	4 (17.4)	1.00, 0.30–3.01	1.000	61 (17.3)
Diabetes mellitus	1 (7.7)	1.84, 0.22–14.49	0.462	0 (0.0)		1.000	2 (40.0)	15.89, 2.51–102.90	0.018	3 (13.0)	3.59, 0.91–13.53	0.079	14 (4.0)
Cigarette smoking	2 (15.4)	0.52, 0.14–2.22	0.527	3 (60.0)	3.88, 0.61–24.03	0.136	3 (60.0)	3.92, 0.61–24.00	0.136	8 (34.8)	1.41, 0.60–3.42	0.475	97 (27.6)

MDR-TB: multidrug resistant tuberculosis (resistance to both isoniazid and rifampicin).

ANY resistance: Resistance to isoniazid, rifampicin, or both; NO resistance: not resistant to either isoniazid or rifampicin.

*Odds ratio estimation not possible due to zero frequency cells.
